# A mixed methods protocol for developing and testing implementation strategies for evidence-based obesity prevention in childcare: a cluster randomized hybrid type III trial

**DOI:** 10.1186/s13012-017-0624-6

**Published:** 2017-07-18

**Authors:** Taren Swindle, Susan L. Johnson, Leanne Whiteside-Mansell, Geoffrey M. Curran

**Affiliations:** 1 Department of Family and Preventive Medicine, University of Arkansas for Medical Sciences, 4301 W. Markham St, #530, Little Rock, AR 72205-7199 USA; 2 Department of Pediatrics, University of Colorado School of Medicine, 12700 East 19th Avenue Box C225, Auora, CO 80045 USA; 3 Department of Pharmacy Practice and Psychiatry, University of Arkansas for Medical Sciences, 4301 W. Markham St, #522-4, Little Rock, AR 72205-7199 USA

**Keywords:** Implementation strategies, Childcare, Obesity prevention, Implementation science

## Abstract

**Background:**

Despite the potential to reach at-risk children in childcare, there is a significant gap between current practices and evidence-based obesity prevention in this setting. There are few investigations of the impact of implementation strategies on the uptake of evidence-based practices (EBPs) for obesity prevention and nutrition promotion. This study protocol describes a three-phase approach to developing and testing implementation strategies to support uptake of EBPs for obesity prevention practices in childcare (i.e., key components of the WISE intervention).

**Methods:**

Informed by the i-PARIHS framework, we will use a stakeholder-driven evidence-based quality improvement (EBQI) process to apply information gathered in qualitative interviews on barriers and facilitators to practice to inform the design of implementation strategies. Then, a Hybrid Type III cluster randomized trial will compare a basic implementation strategy (i.e., intervention as usual) with an enhanced implementation strategy informed by stakeholders. All Head Start centers (*N* = 12) within one agency in an urban area in a southern state in the USA will be randomized to receive the basic or enhanced implementation with approximately 20 classrooms per group (40 educators, 400 children per group). The educators involved in the study, the data collectors, and the biostastician will be blinded to the study condition.

The basic and enhanced implementation strategies will be compared on outcomes specified by the RE-AIM model (e.g., Reach to families, Effectiveness of impact on child diet and health indicators, Adoption commitment of agency, Implementation fidelity and acceptability, and Maintenance after 6 months). Principles of formative evaluation will be used throughout the hybrid trial.

**Discussion:**

This study will test a stakeholder-driven approach to improve implementation, fidelity, and maintenance of EBPs for obesity prevention in childcare. Further, this study provides an example of a systematic process to develop and test a tailored, enhanced implementation strategy.

**Trial registration:**

ClinicalTrials.gov, NCT03075085

## Background

Overweight and obese children are at 5 times greater risk for developing diabetes and at 3 times greater risk for hypertension and high triglycerides in adulthood [1]. Associated health issues include asthma, metabolic risks, depression, and attention-deficit hyperactivity disorder [2, 3]. Socioeconomic disparities in childhood obesity [4] suggest that efforts to prevent excess weight should target contexts serving children at the highest risk.

Given that families impacted by poverty often access childcare through subsidies, childcare provides a critical setting to address socioeconomic disparities in obesity. Further, a typical child will spend 33 h in childcare each week [5] and may eat over half of their dietary intake in this setting, up to 540 meals and snacks per school year [6]. Additionally, nutrition interventions in childcare have been associated with increased willingness to try and liking of new foods [7, 8]. These types of early interventions are key as food habits and preferences are more attributable to environmental factors than genetics [9]. Further, preferences established in early childhood persist across the lifespan [10].

### Suboptimal use of evidence for obesity prevention and nutrition promotion

Despite the potential to reach at-risk children in childcare, current nutrition practices are not consistent with evidence-based obesity prevention [11]. A review of 18 studies in childcare settings found that early childhood educators (ECEs) often do not follow evidence-based nutrition practices, including (but not limited to) signaling hunger cues, avoiding the use of foods for celebration/reward, and allowing children to decide how much to eat without pressure [11].

The underlying reasons for ECE’s poor use of evidence-based practices for obesity prevention and nutrition promotion range from a lack of training related to best practices, lack of policies or poor policy implementation by programs, and ECEs’ personal characteristics and experiences. For example, Sigman-Grant and colleagues [12] documented that the amount of training provided by programs to ECEs was associated with use of evidence-based feeding practices and that characteristics of the training (e.g., type, credentials of trainer) were also associated with variability in uptake of best practices. Yet, only 24% of ECEs in the study received training in this topic more than once per year. Further, a review of state childcare regulations found that agencies vary considerably in their policy efforts to prevent childhood obesity [13], and programs without supportive policy are less likely to use best practices [12, 14]. Finally, personal characteristics of ECEs may be associated with negative practices. Education level and/or race/ethnicity have been associated with pressuring children to finish their food before leaving the table [15], eating less with children, and restrictive feeding practices (e.g., offer food for good behavior) [14]. This evidence suggests that ECEs and childcare centers need additional implementation support for evidence-based obesity prevention.

### Implementation studies to improve adoption of evidence for obesity prevention in childcare

A 2010 systematic review of community interventions failed to identify any trials investigating implementation strategies’ impact on the uptake of evidence-based obesity prevention in childcare [16]. Since this time, Australian researchers have evaluated system-level implementation strategies to promote uptake of environmental changes and policy adoption in childcare. Bell and colleagues [17] found a package of four implementation strategies (e.g., incentives, training, monitoring, and feedback) positively impacted organization-level measures of dietary best practices (e.g., increasing offerings of water and fruit/vegetables). Jones and colleagues [18] tested the impact of a package of eight implementation strategies (e.g., academic detailing, performance monitoring, and feedback) to promote the uptake of seven healthy eating and activity policies in childcare centers. After the trial, centers receiving the package of implementation strategies were more likely than control centers to have implemented two of seven written physical activity and nutrition policies. However, there were no significant changes in child diet or physical activity [19]. Finally, another study testing a single implementation strategy (i.e., educational materials) to promote uptake of nutritional guidelines for menus in Australian childcare found changes in cooks’ intentions to use the guidelines but no changes in centers’ servings of fruits and vegetables [20].

These available studies have focused on system-level adoption of evidence in Australian settings. No studies focusing on ECE uptake of evidence for obesity prevention or nutrition promotion were identified. Further, there is a paucity of research exploring the use of implementation strategies to support uptake of evidence-based practices for obesity prevention in childcare in the USA.

### Aims

In previous research, our research team investigated a “basic” implementation strategy comprised of a 6-h training and monthly reminder newsletters delivered through email in support of a specific evidence-based intervention, WISE (Together, We Inspire Smart Eating) in childcare settings in AR. WISE is an evidence-based obesity prevention intervention to improve ECE feeding practices and children’s dietary behaviors. WISE core components and their supporting evidence base are presented in Table 1.

**Table 1:** Core components of the WISE intervention

Component	Outcomes	References	Type of Evidence
Positive ECE feeding practices (e.g., cues children to hunger, allows food exploration)	Children learn to self-regulate and listen to their body’s cues of satiety. Children are less likely to reject foods and more likely to taste new foods.	[55–61]	RCTs, Quasi-Experimental Trials; American Dietetic Assoc. (ADA) Guidelines; Head Start guidelines
Appropriate role modeling by ECE (e.g., eats healthy foods, talks positively about new foods)	Children are more likely to try new foods and eat healthy foods served.	[60–64]	Quasi-experimental trials, systematic review; ADA guidelines; Head Start guidelines
Multiple, hands-on exposures to fruits and vegetables (FV)	Repeat exposure results in increased intake and liking of FV for children.	[54, 61, 65–70]	RCTs, quasi-experimental trials, systematic review; Head Start guidelines
Use of mascot puppet to promote FV to children	Children are more likely to select and prefer foods associated with familiar characters.	[71–76]	RCT, quasi-experimental trials, systematic review

WISE has shown effectiveness as a total program in time series and non-randomized comparisons [21, 22]. However, findings from the basic implementation support showed that ECE fidelity to WISE components was relatively low. Our definitions of fidelity and the proportions of teachers achieving fidelity at three direct live observations through the school year are provided in Table 2. These data suggest that the basic implementation strategy, while successful for some, only achieved minimally acceptable fidelity for less than half of the participants across the range of components.

**Table 2:** Observed WISE implementation fidelity by core component across a school year (*N* = 44)

WISE component	Fall (month 1) (%)	Winter (month 5) (%)	Spring (month 8) (%)
Role modeling^a^	35.0	43.4	46.7
Use of mascot^a^	15.8	26.0	26.6
Hand-on exposures^a^	57.0	30.3	43.3
Positive feeding practices^b^	30.0	38.2	20.5

^a^Achieving an average score of 3 or better on 1 (not at all)–4 (very much) scale on relevant observed items

^b^On a 1 (never)–4 (4+ times) scale, average supportive feeding practices above 2.5 and unsupportive feeding practices below 1.5 based on observations of lead teachers

The current study aims to understand barriers and facilitators to the uptake of WISE and develop and test additional implementation strategies to improve implementation of WISE in childcare settings in AR. In turn, we predict greater effect sizes on improvements to children’s nutrition and obesity outcomes. Towards these ends, we will complete three aims:

- Aim 1. Identify factors associated with degree of fidelity in a previously developed and tested *basic implementation strategy* of WISE. An explanatory mixed methods approach will use secondary data to identify positive deviance and implementation failures among ECEs in a previous WISE implementation study that observed notable variability in fidelity to best practices for obesity prevention.
- Aim 2. Develop an *enhanced implementation strategy* to support uptake of the WISE intervention using stakeholder input. An evidence-based quality improvement (EBQI) process [23] will be used to engage stakeholders to develop implementation support strategies consistent with an implementation framework (i-PARiHS: integrated Promoting Action on Research Implementation in Health Services) and matched to identified barriers/facilitators.
- Aim 3. Test the impact of the enhanced implementation strategy on implementation and child health outcomes using a Hybrid Type III Cluster Randomized Trial [24] and continuous formative evaluation. We will determine whether the enhanced strategy is feasible, acceptable, and demonstrates improved implementation, fidelity, and sustainability. Further, we will test the hypothesis that better WISE fidelity is positively related to child outcomes (e.g., child fruit and vegetable intake, BMI). A cluster randomization at the center level will be used to avoid cross-over effects among ECEs.

### Theoretical framework

**Fig. 1:**
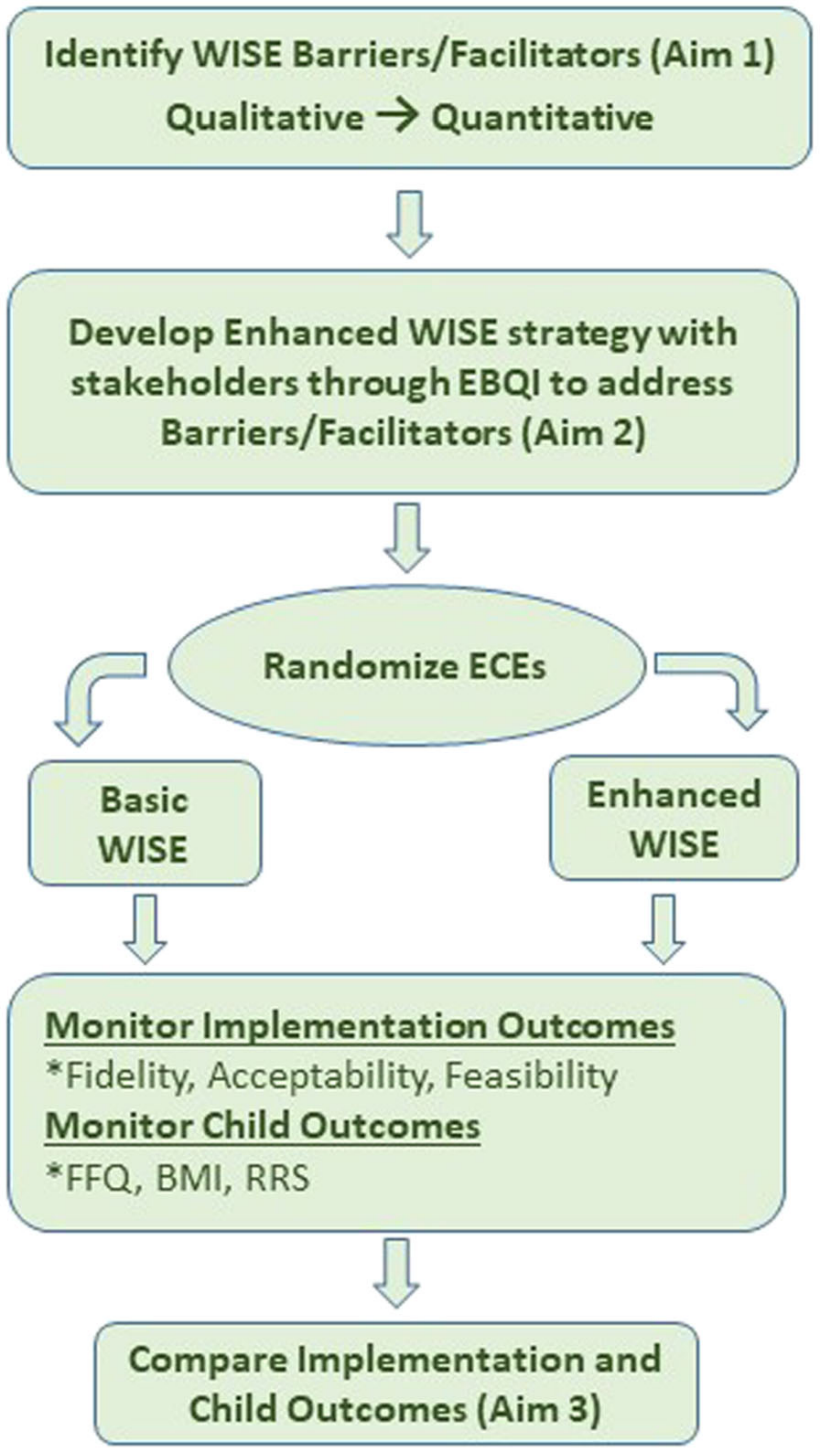
Research aims and design

The i-PARiHS framework guides development of implementation interventions [25]. i-PARiHS posits that core components of successful implementation are innovation (the EB practice), recipients (individual and collective), context (inner and outer), and facilitation. Optimal implementation takes place when facilitation (i.e., implementation strategies) promotes the acceptance and use of an innovation based on recipients’ needs and on the nature of the implementation context. We will use concepts from this framework to identify barriers to implementation of WISE related to ECE characteristics and their childcare settings (Aim 1) and to develop a package of implementation strategies to address barriers (Aim 2). We will then assess the impact of facilitation-based implementation strategies on the success of implementation and subsequent impacts on children and families (Aim 3). Figure 1 summarizes the research aims and design.

## Methods

### Aim 1

#### Study design

We do not yet fully understand why some ECEs were able to achieve fidelity and others were not in the previous implementation of WISE. In this aim, we seek to determine the barriers and facilitators to implementation from the prior study. We will use an explanatory sequential mixed methods design to understand barriers and facilitators specific to WISE implementation (quant → QUAL) [26]. We will also use an approach informed by “positive deviance” methodology. Positive deviants provide insights into facilitators of success and can inform improvements in subsequent interventions. In health care, for example, study of positive deviance has been used to increase the implementation of best practices like hand washing [27]. In our case, we will look at both high and low implementation cases, following the examples of Gabby and colleagues [28] and Rose and colleagues [29]. We expect that positive deviants in previous WISE implementations will be able to identify facilitators that can improve future WISE implementations across contexts. Similarly, we expect that individuals that have been unable to implement WISE with success (i.e., implementation “failures”) will be able to identify previously unknown barriers to implementation of WISE that can be addressed in the enhanced strategies.

Quantitative data gathered from monthly fidelity observations in previous implementations of WISE (i.e., quant) will be used to identify positive deviants and implementation failures for semi-structured interviews (i.e., QUAL). Concepts from the i-PARiHS framework will inform the interview guide. For example, we will ask educators about perceptions of the evidence-based practices that comprise WISE that impede or promote use of the innovation. We will also ask about contextual elements (e.g., values, organizational culture) that made the implementation of WISE easier or more difficult.

#### Participants, setting, and sample size

Previous WISE educators (*N* = 74) with complete observational data will constitute the sample pool for semi-structured interviews. We will also interview directors from each of the 7 sites in previous WISE implementation to provide a multi-stakeholder perspective and expose potential organizational barriers and facilitators. To select educators, we will create a total fidelity score for teachers who participated in the previous study. This score will be a sum of the number of WISE components where fidelity was observed across observations (possible range = 0–32; 4 components × 8 observations). These scores will be used to inform purposive sampling for semi-structured interviews. ECEs with the highest fidelity and lowest fidelity scores will be interviewed. We expect to interview 7 directors and 15 ECEs from each group (*N* = 37). Interviews will last 30–60 min and be transcribed verbatim.

#### Data analysis

Nvivo software will facilitate a shared workspace for the team and aid in organization of coded text. Transcripts will be coded using directed content analysis [30]. The i-PARiHS framework will provide sensitizing concepts upon which to build initial codes. Codes will be used to succinctly label significant, recurrent ideas across participants. To establish reliability, the PI (TS) and a research assistant will code the same two manuscripts. At weekly meetings, the coding pairs will resolve disagreements and expand the codebook. This process will continue until, consistent with standards in team coding, a Kappa of .8 demonstrates inter-rater reliability [31]. After reliability is established, coding will be independent.

### Aim 2

#### Study design

Informed by the barriers and facilitators identified in Aim 1, we will use an Evidence-Based Quality Improvement (EBQI) process [23] to (1) match barriers and facilitators to WISE implementation with potential strategies to improve fidelity to WISE core components, (2) tailor strategies to the early childhood context serving families impacted by poverty, and (3) finalize the enhanced implementation strategy for WISE. The EBQI panel will review the data on the evidence-based strategies, examine the data collected in the formative qualitative interviews, and outline suggested implementation strategies.

#### Participants, setting, and sample size

The EBQI panel will include ECEs and directors from representative sites that will implement the enhanced strategy in the Hybrid Type III trial. We will include at least one teacher from the previous WISE implementation study to have an expert advisor role, providing feedback on feasibility and acceptability of potential enhanced implementation interventions based on prior experiences. We will also recruit parents to inform (a) potential improvements to enhance the link between the classroom and home and (b) strategies to improve assessment of impacts on WISE for future studies. We expect to recruit an EBQI panel of 10 stakeholders and will meet eight times in person or by conference call. We will not recruit stakeholders for the panel that cannot commit to attend a majority of the panel sessions. In the case of an occasional absence, participating stakeholders can nominate a proxy to attend the missed session.

#### Outcomes

After prioritizing barriers and facilitators identified in Aim 1 with the EBQI panel, experts in implementation science on the research staff will review the compilation of implementation strategies from the ERIC project [32] and select options potentially well suited to address the factors of priority considering data on their effectiveness and contexts of previous use. We will then employ a concept mapping approach to invite stakeholders to rate proposed strategies in regards to their importance and feasibility on a Likert scale (1 = not important/feasible, 5 = extremely important/feasible) [33]. This approach provides quantifiable information, promotes efficient collection of input, and provides a basis from which to probe the EBQI panel. We will also record qualitative comments using audio recordings. This process will result in the tailored, enhanced WISE implementation strategy.

#### Data analysis

After each EBQI meeting, the research team will work to assimilate the input from the EBQI panel, translate it to actionable plans, and develop the next iteration of materials for which we seek to receive panel input. We will enter quantitative data (e.g., Likert scale ratings) into SPSS to allow for descriptive statistics across participants. We will also use this information to plot potential strategies on their rated importance (*x*-axis) and feasibility (*y*-axis). Strategies above the mean on both importance and feasibility will be key targets for consideration of the enhanced strategy [33]. For the qualitative information collected from notes and audio recordings, we will employ ongoing coding [34] relative to the main goals of this EBQI process (e.g., matching barriers/facilitators with implementation strategies, tailoring strategies to ECE context). To achieve reliability in coding, primary and secondary coders will code the same manuscripts informed by a start list based on the i-PARIHS framework. At weekly meetings, the coding pairs will resolve disagreements and expand the codebook. Consistent with standards in team coding, [31] Kappa of .8 will be required for coders to code independently with ongoing collaboration to refine codes. We will write memos for each EBQI meeting to inform the development of the enhanced strategy.

### Aim 3

#### Study design

We will use a Hybrid Type III Cluster Randomized Design to test the effectiveness of the enhanced implementation strategy on uptake while also assessing impacts of the intervention on child outcomes. A cluster is defined as a single center at one geographic location. Clusters will be randomized to either (a) the “basic” implementation strategy of WISE or to (b) the “enhanced” implementation strategy of WISE (described below). For both groups, we will collect child outcome data before and after implementation (i.e., dietary intake measures, body mass index). This will provide us with a matched design to assess comparative effectiveness.

Further, we will use formative evaluation principles to assess the feasibility, acceptability, and fidelity of the enhanced WISE strategy during real-time implementation [35]. Formative evaluation is assessment previous to or concurrent with implementation that is used to provide data for immediate use to improve the implementation process *during the study* [24]*.* Gathering implementation-focused formative data will allow us to see trends in this new implementation mode and to make further refinements to the enhanced strategy to arrive at an optimal, fully developed strategy.

#### Participants, setting, and sample size

Head Start is a federally - funded program designed to provide high-quality childcare to low-income families (100% of poverty or below) with children birth to age 5 through from the US Administration for Children and Families Early Childhood Learning & Knowledge Center**.** All centers (*N* = 12) from our partnering Head Start agency will be randomized to implement the basic WISE strategy used in previous studies or the enhanced WISE strategy developed in Aim 2. All centers within the partnering agency are eligible for participation. The number of classrooms in one center ranges from 1 to 10; a total of 41 classrooms are expected. This agency has 760 funded slots and up to this number of children are expected to be included for evaluation of secondary outcomes. All educators in these classrooms will be asked to provide consent for classroom observations. Sites are all with the same county and have a similar demographic makeup of ECEs and families. All families served meet the federal guideline for poverty (e.g., an annual income of $24,250 for a family of 4).

#### Intervention and center randomization

All ECEs will receive basic implementation supports (6-h training at beginning of school year and monthly newsletter). Those in centers randomized to the enhanced condition also will receive stakeholder-informed implementation-support strategies (developed in K01 Aim 2) throughout the year. The enhanced facilitation package will be guided by the work of Waltz et al. on the importance and feasibility of strategies [33], the stakeholders’ ratings of importance and feasibility of strategies (Aim 2), and pragmatic considerations of limiting the package to a parsimonious, sustainable approach. Further, i-PARiHS will inform our selection of strategies for the enhanced package; i-PARiHS emphasizes (and data also support) implementation strategies that either (a) provide recipients with technical skills to do the evidence-based practices (e.g., monitor and feedback) or (b) enable recipients to change their culture or develop their value system (e.g., critical reflection) [36].

A balanced randomization approach will be used to randomize centers to conditions. Center size and key zip code characteristics (e.g., food insecurity, poverty) will be balanced across conditions. This random allocation sequence will be completed by a biostatistician blinded to the study conditions. The PI will inform agency administration who will then distribute training dates to centers based on treatment condition. All ECEs and families served by the agency who consent to research activities after randomization will be included in the study (i.e., complete enumeration). The treatment condition is randomized at the center level and implemented at the classroom level by ECEs. Those randomized to the enhanced condition will receive the additional package of implementation support strategies developed in Aim 2 on a schedule agreed upon by the EBQI panel (e.g., monthly). These supports will be delivered by the PI or a research assistant not involved in outcome measure data collection. To prevent crossover effects, the two groups will be trained separately and asked not to share materials across centers until the completion of the study. The ECEs and data collectors will be blinded to the study condition although the agency administration and investigators will not be.

#### Primary outcomes

RE-AIM provides an evaluation framework to assess key aspects of intervention programs implemented in real-word settings [37]. See Table 3 for a summary of outcome measures that align with RE-AIM and the level of measurement (cluster or individual) for each outcome.

**Table 3:** Outcome measures for hybrid trial

Construct	Measures	Level of measurement
*R*each	Number of children impacted	Cluster
*E*ffectiveness	Child Food Frequency Questionnaire (parent report); child Body Mass Index (record review); child Resonance Raman Spectroscopy scan	Individual
*A*doption	Food purchase records reflecting the number of WISE lessons completed; Resources distributed, Organizational Readiness to Change Assessment	Cluster; individual
*I*mplementation	WISE fidelity, acceptability, feasibility	Individual
*M*aintenance	Proportion of ECEs maintaining/ increasing in fidelity after 6 months	Cluster

*Reach* will be reflected by teacher report of the number of realized opportunities for WISE lessons divided by the number of possible opportunities (target = 4 opportunities per child per month). *Adoption* will be measured using teacher reports of the number of WISE parent handouts/recipes on target foods distributed (target = 2 per child per month), teacher report of the number of WISE lessons and activities presented each month, and assessment of food purchase records to assess frequency of purchase of WISE foods. Further, we will modify and use The Organizational Readiness to Change Assessment (ORCA) [38] as developed for use with the i-PARiHS framework to assess change commitment (e.g., We value this change) and change efficacy (e.g., We can keep the momentum going) both prior to and during implementation.

For *Implementation*, a WISE fidelity measure will be used across the school year. The WISE fidelity instrument is rated on a 1 to 4 scale with 4 representing the highest level of fidelity. Each core component is assessed with 2 items. Average fidelity scores above 3 are considered to reflect adequate fidelity on a component. Additionally, overall scores on the fidelity form are created by summing scores across items (range = 0–32). Inter-rater reliability of 85% will be ensured. Finally, acceptability and feasibility [39] will be assessed through semi-structured interviews at two time points: (1) between the fall and winter and (2) winter and spring fidelity assessments. *Maintenance* will be assessed by determining the proportion of teachers that increase or remain the same in adoption and fidelity from the initial assessment (fall) across the school year (winter and spring). We will complete fidelity observations three times per year: fall (Sept–Oct), winter (Jan–Feb), and spring (March–April). We will train contract staff to conduct fidelity assessments using videos of previous WISE lessons and ensure 85% reliability is achieved.

#### Secondary outcomes

In addition, we will include outcome measures related to impacts of the program on children (i.e., *Effectiveness*). These secondary outcomes will include parent report of child dietary consumption, child Body Mass Index (BMI), and biomarkers of carotenoid intake through resonance Raman spectroscopy (RRS). All families complete a Family Map Inventory (FMI) [40] for fall and spring in an interview with the ECE of family strengths and needs. The FMI is a standard part of programing for this Head Start agency. For this study, the FMI will include a modified, qualitative Food Frequency Questionnaire (FFQ) to assess consumption of WISE foods. The format reflects response options as given in the Fred Hutchinson FFQ forms [41] and focuses on WISE foods only. Parent completion of FFQ has been validated against blood serum levels in previous studies and is effective for identifying children in the highest and lowest quartiles of intake for specific nutrients (i.e., effective for large-scale surveillance). [42] BMI is a required twice-yearly, federal assessment for Head Start children. An anonymous record review of these data will provide a comparison of impacts on child diet between the basic and enhanced implementation conditions.

We will collect (RRS scans from children with equipment leased from NuSkin. RRS is a promising alternative for measuring biomarkers where carotenoid levels are measured by an optical scan of the palm [43, 44]. It has been used safely in prior studies with children aged 3 and older. Carotenoids (i.e., plant pigments) are phytochemicals that provide the bright colorings to vegetables [45]. When ingested, carotenoids become biomarkers for dietary habits, evident in the makeup of cell tissues including the skin [46]. RRS measurements are reflective of dietary intake over the previous 4 weeks. RSS scans are sensitive to detecting individual differences of carotenoid levels [47, 48] and experimentally initiated changes [49, 50]. We expect to collect this information from up to 760 children between the ages of 3 and 5.

#### Formative evaluation

Measures gathered throughout the year will be used to continue improvements of the enhanced implementation strategy (i.e., formative evaluation). In addition, after the fall and winter assessments, we will determine which teachers are achieving fidelity. We will randomly select 5 ECEs to complete semi-structured interviews with study staff on aspects of feasibility and acceptability of the implementation. After analysis of these interviews at each of the two iteration points during the school year, we will hold EBQI meetings to review the themes that emerged and the observed fidelity in the classrooms up to that point. With feedback from the EBQI panel, we will use this information to determine shifts needed to improve the enhanced strategy for the remainder of the school year.

#### Data analysis and power

Ongoing descriptive analyses of outcome measures will be conducted to inform formative evaluation. At the completion of data collection, analyses of each RE-AIM outcome measure will be examined by descriptive statistics to determine outliers. Multi-level models (MLM) will be used to account for the dependence among repeated observations of the same teachers in the same classroom as well as children nested within classrooms. The MLM models will include a fixed term for intervention (basic versus enhanced) and time. Random effects will be included for the correlation of children within classrooms and the correlation of observations within teacher. This analysis will allow for estimation of variance in child outcomes accounted for by implementation (i.e., level 2) effects.

We used data from the previous WISE study to estimate the variability in outcomes that is accounted for by nesting in center and class at baseline. Our power analyses accounted for the three levels of nesting, students nested in classrooms, nested in centers with treatment assigned at the center level. With 10% of variance in the child outcomes accounted for by covariates (e.g. ethnicity, gender), 1% of the variance accounted for by nesting in center, and 3% of the variance accounted for by nesting in classroom, our design will provide us with 80% power to detect and effect of .40 (a half standard deviation) given a two-tailed test with alpha set at 0.05.

For formative evaluation interviews with educators, we will complete rapid coding [34] of the semi-structured interviews with the selected ECEs at each iteration (i.e., Sept–Oct and Jan–Feb). Interviews will focus on soliciting ECE opinions on what is working or not working about the enhanced implementation strategy to support their uptake of the WISE components and what changes they would suggest. The schedule of iterations will allow 2 months between measurement occasions to complete coding, consider the information with the EBQI panel, and to design improvements to the enhanced strategy. Coding will focus on identifying factors most anticipated to impact implementation feasibility and acceptability. Two coders will complete initial content analysis independently and come together to resolve any differences.

## Discussion

This protocol described above proposes unique research expected to be one of the first studies in the USA to examine the impact of implementation strategies on outcomes of an evidence-based intervention for obesity prevention in childcare. In the field of Implementation Science, Proctor and colleagues [51] call for moving beyond studies of implementation barriers to build and test implementation strategies. This study answers this call by using a systematic stakeholder-driven process to develop and pilot test a customized “enhanced” implementation strategy compared to an “implementation as usual” strategy. We believe this will be the first study to employ the EBQI process to inform community, rather than clinical, implementation interventions.

### Challenges and limitations

A recent synthesis delineated a staggering 73 distinct implementation strategies for consideration (with varying evidentiary support) [32]. To narrow this list, we have described a process that will consider our theoretical framework, the evidence base on implementation strategies, and the stakeholders’ opinion of potential strategies. These guiding sources will focus our efforts and promote efficient selection of strategies. An additional challenge will be ensuring continued engagement of stakeholders. To address this challenge, we will engage the leadership at each site by inviting them to be on the EBQI panel. This has been associated with increased buy-in in previous EBQI efforts [52].

### Conclusion

By using a Hybrid Design, we will add to the literature on the preventive effectiveness of WISE while exploring how obesity/nutrition outcomes vary by implementation fidelity. If the enhanced strategy outperforms the “basic” strategy for the adoption of best practice and impacts on children as hypothesized, this will provide evidence that the additional resources required to support implementation of EBIs (evidence-based interventions) are warranted. Our study will also provide an example of how Implementation Science concepts such as Hybrid Designs can be applied to study of community-based nutrition interventions. Ultimately, improvements in evidence-based obesity prevention in childcare have the ability to impact 11 million children under age 5 in the USA annually [53].

## Abbreviations

AR: Arkansas
BMI: Body Mass Index
EBQI: Evidence-based quality improvement
ECE: Early childhood educators
FFQ: Food Frequency Questionnaire
FMI: Family Map Inventory
i-PARiHS: Integrated Promoting Action on Research Implementation in Health Services
IRB: Institutional Review Board
MLM: Multi-level models
ORCA: Organizational Readiness to Change Assessment
RRS: Resonance Raman Spectroscopy
UAMS: University of Arkansas for Medical Sciences
WISE: We Inspire Smart Eating
